# Bridging historical antigenic profiling and whole-genome taxonomy in *Rickettsia*

**DOI:** 10.1038/s44573-026-00004-2

**Published:** 2026-09-14

**Authors:** Aticha Lhokaew, Elizabeth M. Batty, Stuart D. Blacksell

**Affiliations:** 1 Mahidol Oxford Tropical Medicine Research Unit, Faculty of Tropical Medicine, Mahidol University, Bangkok, Thailand; 2 Centre for Tropical Medicine and Global Health, Nuffield Department of Medicine, University of Oxford, Oxford, UK

**Keywords:** *Rickettsia* taxonomy, *Rickettsia* group antigenic profile, Serological diagnosis, Whole genome sequencing, Computational biology and bioinformatics, Evolution, Genetics, Microbiology

## Abstract

Historically, rickettsial diseases were classified primarily through antigenic profiling and serotyping, an approach that was later superseded by molecular taxonomy and phylogenetics. Recent advances in whole-genome sequencing (WGS) have revealed substantial taxonomic ambiguity within the genus *Rickettsia*, including nomenclatural redundancy and inconsistent species delineation, raising questions about the adequacy of current classification criteria for obligate intracellular bacteria. In parallel, long-standing serological data demonstrate extensive, structured antigenic relationships that have not been fully explored within modern genomic frameworks. In this review, we re-examine historical antigenic profiling in the context of contemporary WGS-based taxonomy and concordance between serological cross-reactivity and genetic relatedness across *Rickettsia* groups, particularly within and between the spotted fever, transitional, and typhus groups. Integrating antigenic and genomic data provides a polyphasic perspective that may aid in resolving current taxonomic uncertainty and has direct implications for serological diagnosis, antigen selection, and future taxonomic proposals. We argue that revisiting antigenic data alongside genomic metrics is essential to developing a coherent, biologically meaningful framework for *Rickettsia* classification.

**Supplementary Information:**

The online version contains supplementary material available at https://doi.org/10.1038/s44573-026-00004-2.

Rickettsial diseases are among the most common emerging neglected diseases in tropical areas and worldwide, posing the highest risks to those in poverty and marginalised communities. This prevalence is partly due to their arthropod vector-borne nature and the difficulty of diagnosing rickettsial diseases because of their nonspecific febrile clinical presentation. Considered one of the most common acute febrile illnesses after dengue and often underdiagnosed^1–3^, rickettsial diseases pose a significant disease burden worldwide, but current burden estimates are presumed to be inaccurate and understated due to limited data^4^.

Historically delineated by mouse serotyping and antigenic profiling, the Rickettsiales as a whole have had a long-standing history of taxonomic and delineation inconsistencies. This ambiguity was addressed as early as 2001 by Dumler et al., playing a crucial role in the molecular reorganisation of Rickettsiales and Anaplasmataceae, whose work now serves as the basis of present-day taxonomy^5,6^. Recent whole-genome sequencing studies have revealed substantial ambiguity in current Rickettsia species definitions, raising questions about how species should be delineated in an obligate intracellular genus with limited phenotypic diversity^7,8^. At the same time, extensive historical antigenic profiling data, often regarded as obsolete with the advent of the genomic era, capture structured biological relationships that remain standard in serological assays such as IFA, the gold standard for *Rickettsia* diagnosis^9,10^.

Recent years have seen renewed efforts to reconcile existing *Rickettsia* taxonomy with genomic methodologies; however, traditional bacterial species delineation guidelines yield drastic amendments to pre-existing taxonomy^5,8^. Two notable factors exacerbate divergence between pure WGS taxonomy and traditional taxonomy in *Rickettsia*: firstly, the disregard of crucial polyphasic characteristics such as geographical and phenotypic traits; secondly, the obligate intracellular nature of *Rickettsia* results in a smaller genome, reductive evolution and high synteny^11^. As such, current whole-genome sequencing-based classification of *Rickettsia* has exposed substantial taxonomic ambiguity that cannot be fully resolved by genomic metrics alone in an obligate intracellular genus with reduced phenotypic diversity.

The historical antigenic profile remains an important facet of Rickettsiology and houses important phenotypic insights valued in the modern polyphasic species delineation approach. This review investigates historical serological evidence alongside contemporary genome-based taxonomy to assess their concordance and propose a polyphasic framework for resolving rickettsial classification and diagnostic challenges. Here, we synthesise historical antigenic and serological data with contemporary genome-based phylogeny, demonstrating that early antigenic groupings broadly correspond to many relationships recovered by modern WGS analyses, and argue that reintegrating these data is an important pillar for coherent taxonomy and rational serological diagnostic design.

## Overview of *Rickettsia* taxonomy and phylogenetic lineages

### General *Rickettsia* group classification

The genus *Rickettsia* was named after Howard Taylor Ricketts for his 1906 research on the causative agent of Rocky Mountain spotted fever, *Rickettsia rickettsii*. Currently, *Rickettsia* bacteria are broadly classified as members of the genus *Rickettsia*, though colloquially the term “*Rickettsia*” encompasses the Order Rickettsiales, which includes a diverse group of obligate intracellular Gram-negative bacteria most commonly associated with arthropods such as ticks, lice, fleas, and mites. The Order Rickettsiales includes three main family lineages: Rickettsiaceae, Anaplasmataceae, and Candidatus Midichloriaceae. Outside of the genus *Rickettsia*, other genera found within Rickettsiales include *Orientia*, *Wolbachia*, *Anaplasma*, *Ehrlichia*, and *Neorickettsia*. Over 30 described *Rickettsia* spp. belong to the genus *Rickettsia*, alongside many candidate species and species known only as tick endosymbionts.

**Fig. 1:**
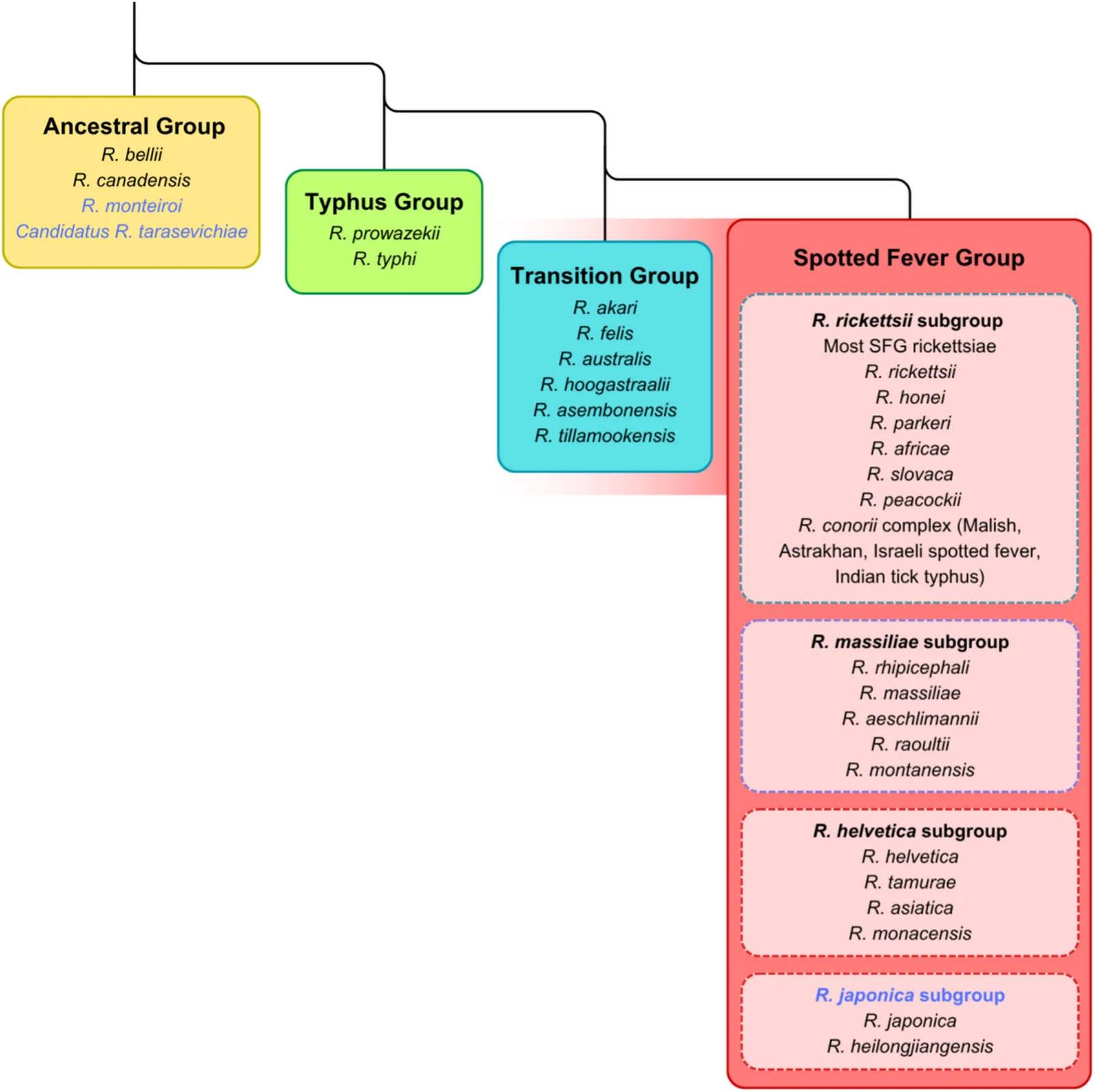
Schematic cladogram of *Rickettsia* spp. categorised into groups based on prior understanding of *Rickettsia* phylogeny^12–14,108–112^. TRG is considered part of SFG in much of the literature, particularly antigenically (Blue font indicates inconclusive, debated, or unconfirmed placement within a group or debated group status).

As obligate intracellular arthropod-borne Gram-negative bacteria of the Order Rickettsiales, *Rickettsia* species can be classified into four primary groups: Spotted Fever Group (SFG), which includes *Rickettsia conorii* and *R. rickettsii*, Typhus Group (TG), which includes *Rickettsia prowazekii* and *R. typhi*, Transitional Group (TRG), which includes *Rickettsia felis*, and Ancestral Group (AG), which includes *Rickettsia bellii* (Fig. 1). Ongoing taxonomic confusion plagues the genus, particularly the question of what constitutes a *Rickettsia* species. Past serotyping was heavily utilised to characterise *Rickettsia* species before gradually phasing out as advances in genetic research came to prominence. However, serotyping and subsequent advances in *Rickettsia* serology may still help reconcile our understanding of *Rickettsia* taxonomy as we know it.

### Spotted fever group (SFG)

The largest and best-known group within the *genus Rickettsia* is SFG, which includes most *Rickettsia* spp. such as *R. rickettsii*, and *R. conorii*, the causative agent of Mediterranean spotted fever (Table 1). Pathogenic SFG bacteria cause febrile rashes or spotted fever, hence their namesake. Within SFG, phylogenetic clades have been established using multigene approaches to infer phylogenetic relationships, initially relying on several well-known rickettsial markers, including 16S rRNA, *htrA*, *glt*A, *omp*A, *omp*B, *sca*4, *sca*1, and *sca*2^12^. Later, whole-genome analysis was utilised instead for a more comprehensive phylogenetic overview^13^. To summarise these informal subgroups broadly, three have been described: the *Rickettsia massiliae* subgroup, the *Rickettsia helvetica* subgroup, and the *R. rickettsii* subgroup^12–14^. Additionally, the *Rickettsia japonica* subgroup (referred to as *R. heilongjiangnensis* subgroup in this literature) has been briefly noted, though this classification remains under debate^12,13^.

**Table 1:** Representative list of validated and unvalidated SFG species without formal selection criteria to illustrate the vast number of species within the group. An asterisk (*) after the species name indicates a species with limited data.

Spotted Fever Group Species			
Species	Disease or Clinical Manifestation	Vector	References
*R. rickettsii*	Rocky Mountain spotted fever	Tick	^73^
*R. conorii*	Mediterranean spotted fever, Astrakhan fever, Israeli spotted fever, Indian tick typhus	Tick	
*R. parkeri*	*R. parkeri* rickettsiosis	Tick	
*R. rickettsiI* subsp. *californica* (formerly *R. philipii*; previously known as *Rickettsia* sp. 364D)^80^	Pacific Coast tick fever	Tick	
*R. africae*	African tick bite fever	Tick	
*R. japonica*	Japanese spotted fever	Tick	
*R. heilongjiangensis*	Far-Eastern spotted fever	Tick	
*R. honei*	Flinders Island spotted fever	Tick	
*R. amblyommatis*	Mild spotted fever or asymptomatic	Tick	
*R. sibirica*	Siberian tick typhus	Tick	
*R. slovaca*	Tick-borne lymphadenopathy	Tick	
*R. raoultii*	Tick-borne lymphadenopathy	Tick	
*R. helvetica*	Mild self-limiting disease, but can become severe	Tick	^34,93,94^
*R. monacensis*	Mild spotted fever	Tick	^95^
*R. aeschlimannii*	Mild spotted fever	Tick	^96^
*R. massiliae*	Mild spotted fever, potential to become severe	Tick	^97^
R. asiatica *	-	Tick	
R. cooleyi *	-	Tick	
R. martinet *	-	-	
*R. mongolotimonae*	Acute Rickettsiosis	Tick	^98,99^
*R. hulinensis/hulinii*	Potentially acute rickettsiosis in humans	Tick	^99–101^
*R. montanensis*	Afebrile Rash	Tick	^102^
R. rhipicephali *	-	Tick	
R. sharonii *	Israeli tick typhus	Tick	^19^
*R. tamurae*	Local inflammation	Tick	^103^
*R. peacockii*	-	Tick	^104^
R. argasii *	-	Tick	
*R. fournieri*	-	Tick	^105^
*R. lusitaniae*	-	Tick	^106^
R. thailandii *	-	Tick	
R. moreli *	-	Tick	

### Transitional group (TRG)

A fourth subgroup within SFG is historically the *Rickettsia akari* subgroup, with notable initial members including *R. felis* and *R. akari*. Through genetic analysis of this subgroup, the affinity between *R. felis*, *R. akari*, and AG rickettsiae was first elucidated, based on the close relationship between the putative conjugative plasmid (pRF) in *R. felis* and AG *Rickettsia* genomes, supporting the subsequent proposal of TRG as a separate clade of *Rickettsia*^12,15^. However, some authors may still prefer to group TRG within SFG because their differences are mainly genetic, and they, by and large, retain antigenic similarities with SFG^16,17^. In either classification, the TRG is generally considered valid. An analysis in 2022 also proposed a new classification of SFG and TRG into SFGI and SFGII, respectively and placed *R. helvetica* basal to TG but not to SFG entirely^11^.

### Typhus group (TG)

TG was initially separated from SFG based on antigenic profile, serological cross-reactivity, clinical differences, and vector species^12,18,19^. Although the TG phylogenetic group includes only two species, *R. prowazekii* and *R. typhi*, members of this group have been clinically important to humans for centuries, particularly *R. prowazekii*, which has had a long impact throughout human history as causing the plague epidemic typhus that follows in the wake of wars, famine, and poor living conditions^20^.

### Ancestral group (AG)

Lastly, AG is the basal lineage of all *Rickettsia* members as the earliest rickettsiae to have diverged from SFG and TG. *Rickettsia bellii* is the first to be characterised as neither a member of SFG nor TG, but rather a member of a group that diverged before the separation of SFG and TG Rickettsiae, basal to both groups^21^. AG includes only two members: *R. bellii* and *Rickettsia canadensis* (formerly *Rickettsia canada*). Notably, El Karkouri et al. proposed *R. canadensis* and *R. bellii* be re-classified as separate Canadensis and Bellii groups, respectively, due to their basal placement on separate external phylogenetic branches^11^, which may shape future classification of AG moving forward.

## Historical and contemporary approaches to rickettsial classification

### Historical methods of rickettsial classification

Species in the genus *Rickettsia* were first classified into antigenic groups based on serology, particularly mouse serotyping, leading to the SFG and TG groups^22,23^. Subsequent increased awareness of phenotypic characteristics, including epidemiological, antigenic, and immunological properties, as well as genetic characteristics, led to the current four groups of TG, SFG, TRG, and AG^8,12,15,21^.

### Transition to molecular phylogenetics

Before the widespread adoption of whole genome sequencing (WGS) for elucidation of phylogeny and species designation, classification of bacteria initially relied on DNA G + C content and DNA-DNA hybridisation (DDH) in the 1960s, followed by the widespread popularisation of 16S rRNA taxonomic classification, which was considered a gold standard in phylogenetic reconstructions^24,25^ for a long time. Currently, improvements in 16S rRNA classification are achieved by typing multiple conserved genes in *Rickettsia* and classifying species based on sequence similarity^26,27^. The most used scheme combines pan-bacterial *16S rRNA*, *gltA*, and SFG rickettsial-specific *OmpA* (*sca0*), pan-rickettsial *OmpB* (*sca5*), and pan-rickettsial *gene D* (*sca4*), with the rationale that OmpA and OmpB are large, immunodominant surface proteins with substantial genetic variation between species^28–34^. Under this scheme, a putative *Rickettsia* candidate must have ≥ 98.1% homology with *16S rRNA* and > 86.5% with *gltA* to an existing *Rickettsia* species. Generally, multiple gene schemes provide greater phylogenetic resolution than *16S rRNA* alone, owing to the presence of multiple metabolic genes. Within the *Rickettsia* genus, both *16S rRNA* and multiple-gene-derived phylogenies have been shown to strongly agree with the WGS phylogeny^8,35^. A ribosomal multilocus sequence typing (rMLST) scheme^36^ that uses 53 conserved ribosomal genes to distinguish between *Rickettsia* species has not been widely used.

## Whole genome sequencing and the taxonomic realignment of *Rickettsia*

### Introduction of whole genome metrics and rickettsial complexity

**Fig. 2:**
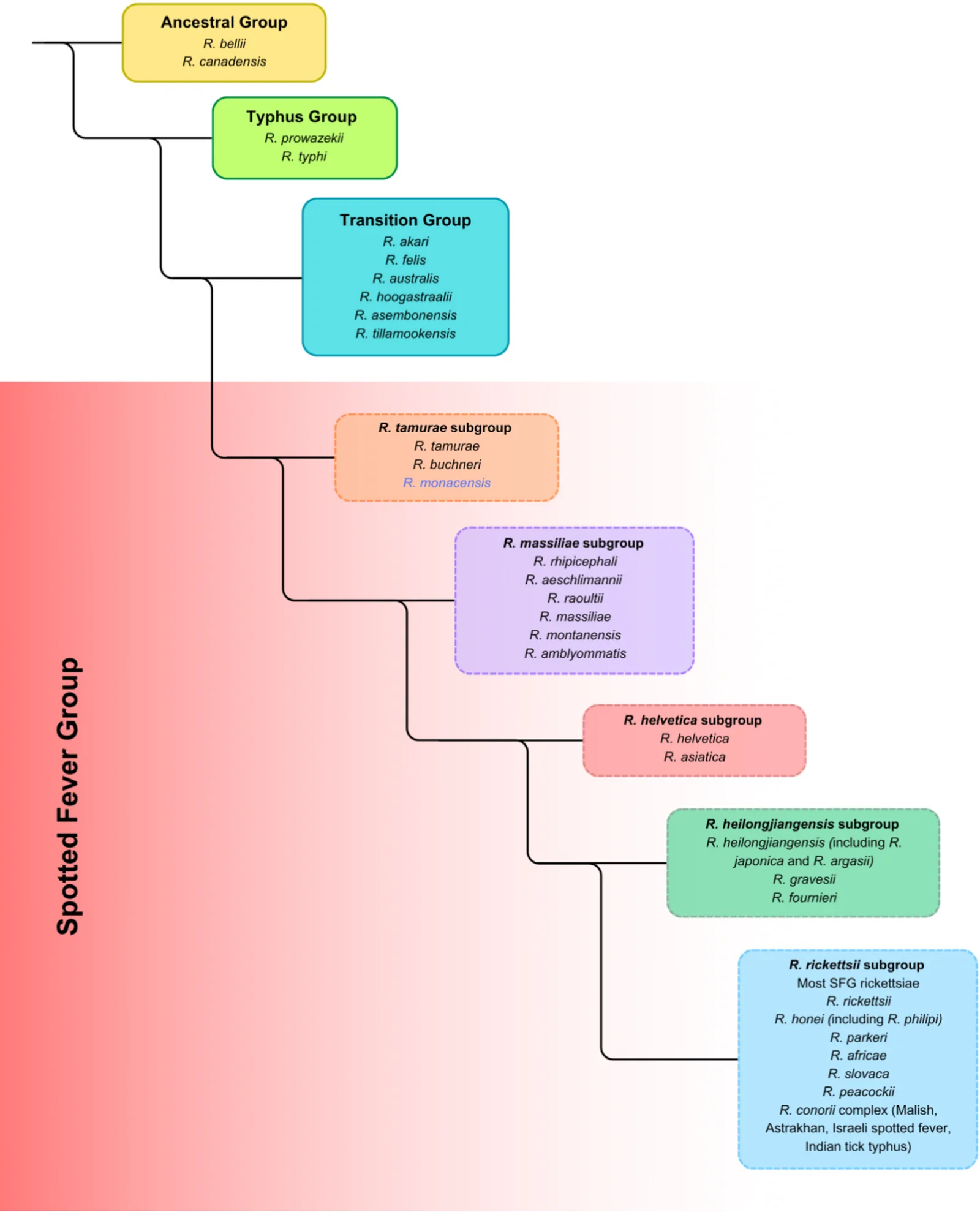
Conceptual schematic summarising broad phylogenetic and antigenic relationships among major Rickettsia groups discussed in this review. The figure integrates published whole-genome phylogenies and historical antigenic relationships. Branch lengths, node positions, and subgroup boundaries are illustrative only and do not represent evolutionary distance or a formal phylogenetic reconstruction. (Blue font indicates inconclusive, debated, or unconfirmed placement within a group.)

Recent literature utilising WGS^5,8,35^ provides further clarification on the *Rickettsia* phylogeny as well as argues for species designation revision, finding several taxonomic heterotypic synonyms and making the case that several species should instead be designated subspecies to other existing species, while others expand into *Rickettsia* genetic evolution and diversity, proposing new phylogenetic groupings^11^. We have summarised this observed relationship with a conceptual schematic in Fig. 2. The reason for this renewed scrutiny is the lack of an appropriate taxonomic system for *Rickettsia* and the lack of significant clinical and phenotypic data^5^. A noteworthy issue repeatedly raised in the existing WGS literature is the problematic and inconsistent nature of *Rickettsia* delineation and nomenclature, whilst *Rickettsia* monophyly and taxonomy are supported. In fact, the topic of rickettsial taxonomy criteria has long been contentious within the field^5,7,37^. Outside the field, large-scale analysis of bacterial whole genomes is used to define genus and species boundaries, and these automated classifications, based on thresholds across the whole bacterial taxonomy, are often inconsistent with historical classifications or with those defined by whole-genome sequence analysis within the Rickettsial genus^5,35^. Genomic data is currently available for 86 species or subspecies as recognised by NCBI, including eight candidatus species. A summary of public *Rickettsia* genome sequences deposited in NCBI Genbank is presented in Table S1.

### Breakdown of current WGS-based taxonomic tools and thresholds

WGS-based taxonomic metrics rely on various methods to quantify the overall genome-relatedness index (OGRI). The most well-known and widely adopted bacterial species delineation methods are DDH/digital DDH (dDDH), average nucleotide identity (ANI), and genome blast distance phylogeny (GBDP)^38^. DDH traditionally measures genetic distance between two organisms and is used extensively in bacterial species delineation and phylogeny, with dDDH subsequently developed as a bioinformatic in silico analogue^39^, whereas ANI measures nucleotide similarity between genomes, with its later derivative orthoANI, which modifies ANI to use orthologous fragments and be reciprocally consistent^40^. Together, dDDH and ANI are typically used to delineate bacterial species. Other metrics, such as average amino acid identity (AAI) and core genome alignment sequence identity (CGASI), have also been implemented. CGASI uses the sequence identity of a concatenated core genome alignment to infer phylogenetic relationships. AAI determines genomic relatedness through amino acid identity and is especially preferred beyond species rank, as methods such as ANI are less useful for more distantly related genomes.

### *Rickettsia*-specific WGS threshold revisions and species merger proposals

Major taxonomic resources used in bacterial genetics do not match the species groupings used in rickettsial research. The National Center for Biotechnology Information (NCBI) Taxonomy database is used to classify nucleic acids submitted to any International Nucleotide Sequence Database Collaboration (INSDC) database. Under the NCBI taxonomy^41,42^, all TRG species are grouped into the SFG, *R. raoultii* and *R. heilongjiangensis* are subspecies of *R. conorii*, and *R. buchneri* is a subspecies of *R. tamurae*. The Genome Taxonomy Database^43^, designed to provide a standardised taxonomy for improved classification of novel bacterial species, uses a 95% ANI cutoff, which reclassifies most SFG species as *R. rickettsii.*

**Fig. 3:**
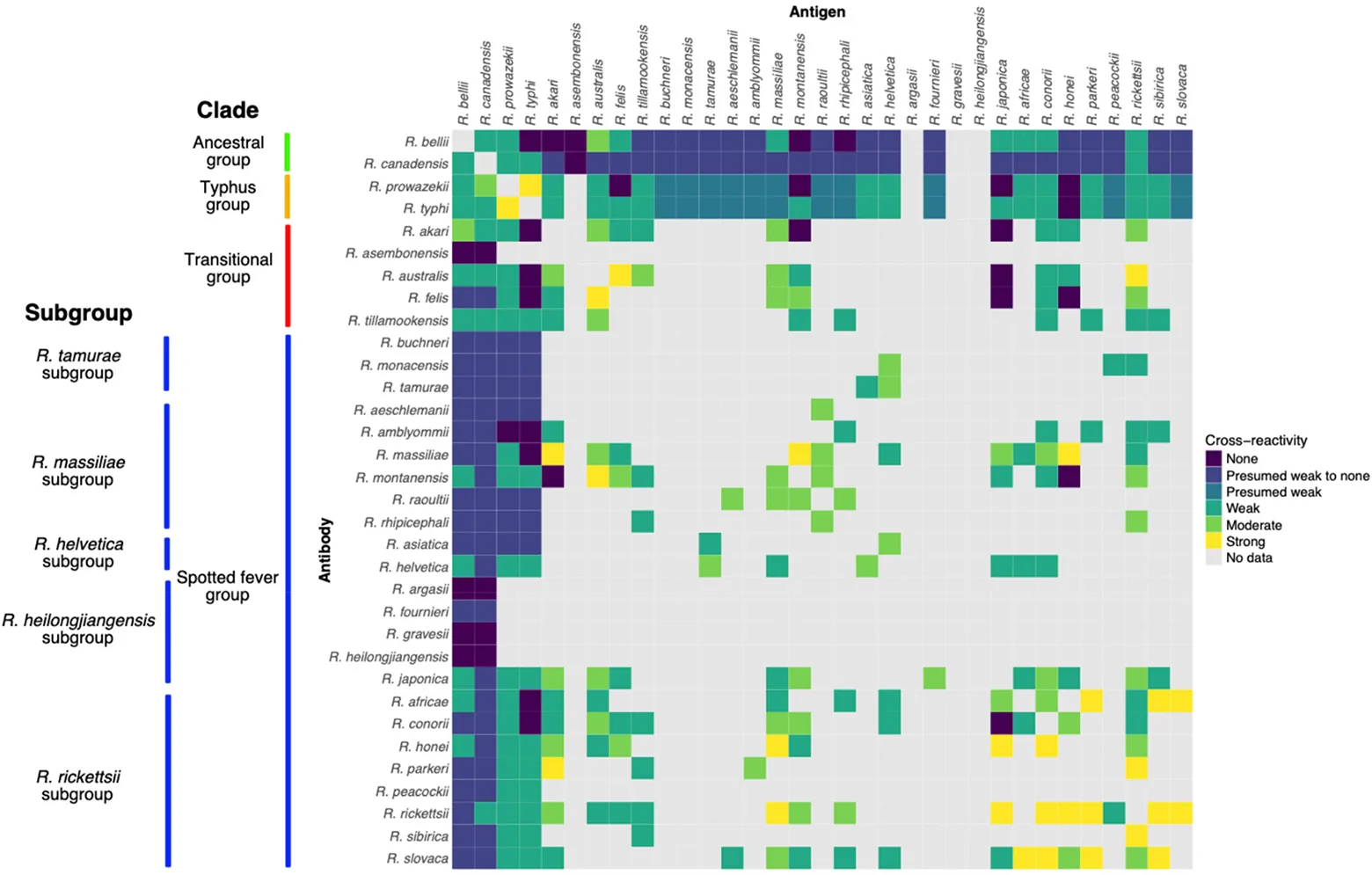
A heatmap showing serological cross-reactivity between different *Rickettsia* species, grouped by our proposed clade and subgroup as shown in Fig. 2. Data taken from papers cited in this review, alongside additional data^104,113–125^. “No data” is shown in grey where no serological cross-reactivity data source was available.

**Fig. 4:**
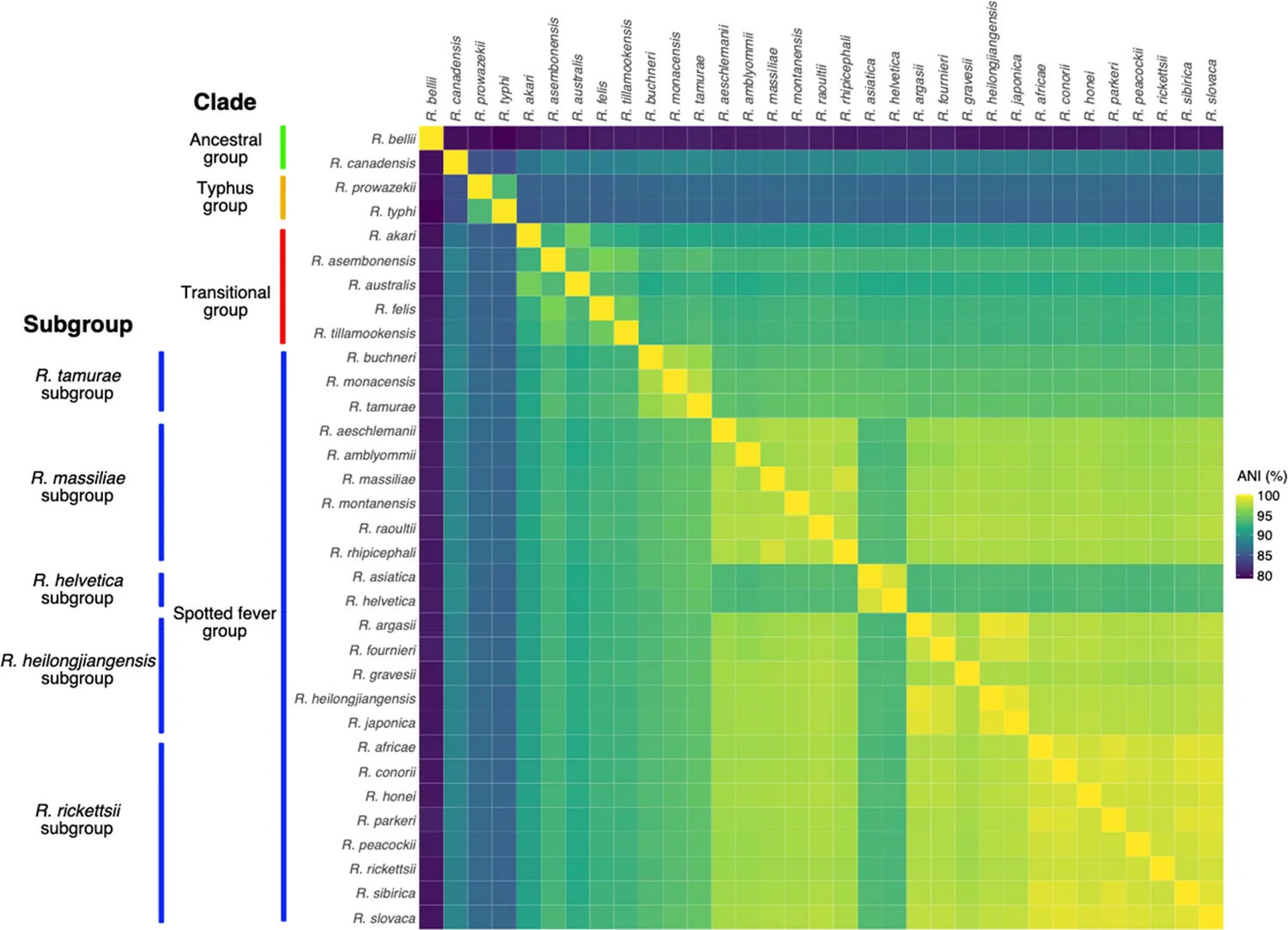
A heatmap showing average nucleotide identity (ANI) between different *Rickettsia* species, grouped by our proposed clade and subgroup as shown in Fig. 2. Reference genomes for each strain are listed in Table S2, and ANI was calculated using FastANI, using the matrix values for each comparison.

Genus-specific analyses have proposed alternative criteria for species delineation in *Rickettsia* (Table 2). Diop et al.^8^. proposed a modified criterion of both ANI and dDDH, as the current ANI of ≥95–96% and a dDDH value of ≥70% is inapplicable to *Rickettsia* spp. based on *Rickettsia’s* few phenotypic properties and low genetic heterogeneity, coupled with its obligate intracellular lifestyle. Doing so would merge several species into a single species, such as *R. canadensis* with *R. bellii*, *R. typhi* with *R. prowazeki*i, and the entire TRG. Chung et al.^5^. also demonstrated that the equivalent CGASI will classify the majority of SFG as the same species, except for *R. monacensis*, which is classified into a single species. Hördt et al*.*^35^ utilised a dDDH cutoff of 70%, and the same issue arises: *R. gravesii*, *R. heilongjiangensis*, *R. japonica*, *R. sibirica*, and *R. slovaca* are classified as *R. conorii* subspecies, and *R. buchneri* is classified as an *R. tamurae* species. As such, Diop et al. proposed a new species demarcation criterion of >92.3% dDDH and >99.19% orthoANI, respectively. Under these revised *Rickettsia*-specific criteria, only *R. heilongjiangensis* and *R. japonica* are to be assigned together at 92.4% dDDH according to Hördt et al. According to Diop et al., *R. argasii should* be reassigned to *R. heilongjiangensis*, and *R. rickettsii* subsp. *californica* (formerly *R. philipii*; previously known as *Rickettsia* sp. 364D) should be reassigned to *R. rickettsii*. Additionally, other *Rickettsia*, such as *R. sibirica* and *R. parkeri*, with notably high dDDH and orthoANI values of 92.3% and 99.19%, respectively, are observed and serve as the dDDH and orthoANI cutoffs in the paper. There is also the possibility that this criterion could paradoxically be too lax, as Chung et al. note in their own CGASI calculation: the genus-species delineation for *Rickettsia* is currently problematic and should be excluded from the analysis. In which case, Diop et al.’s decision to base species demarcation cutoffs on the current list of validly published species as the gold standard serves as a pragmatic solution for the time being. However, using high dDDH and orthoANI cutoffs chosen to separate *R. sibirica* and *R. parkeri* raises the question of whether they are sufficiently distantly related to constitute separate, distinct species. Across all three WGS datasets, however, a consistent phylogenetic tree pattern is observed with consistent groupings, especially in SFG (Figs. 3 and 4, Supplementary Table S2).

**Table 2:** Table summarising current WGS-based taxonomic thresholds in *Rickettsia* species delineation.

Taxonomic tool/ORGI	Criteria for species delineation	References
Traditional dDDH and ANI	ANI of ≥95–96% and a dDDH value of ≥70%	^35,38,107^
Proposed revised ANI and dDDH for *Rickettsia*	To qualify as a *Rickettsia*: OrthoANI value of ≥83.63 % To qualify as a new *Rickettsia* species: >92.3% dDDH and >99.19% orthoANI	^8^
CGASI	CGASI value of ≥96.8%	^5^
Past Multigenic *Rickettsia* Scheme (non-WGS)	To qualify as a *Rickettsia*: ≥98.1 of 16S rRNA homology and ≥86.5% of *gltA* homology. To qualify as a new *Rickettsia* species: ≥99.8 of 16S rRNA homology; ≥99.9% of *gltA* homology; ≥98.8 of *ompA* homology, ≥99.2 of *ompB* homology, and ≥99.3% of *gene D* homology.	^34^

## Antigenic structure and group identity of *Rickettsia* spp.

The taxonomic uncertainty revealed by WGS raises the question of whether historical antigenic data, used to define rickettsial groups long before genomic tools were available, capture biologically meaningful relationships. While WGS-based taxonomic techniques are powerful and discriminatory, current species characterisation emphasises a comprehensive polyphasic approach, considering not only genetic similarity but also bacterial characteristics such as geographical, chemotaxonomic, and phenotypic traits^44,45^. One such important characterisation, specifically within the genus *Rickettsia*, with a long historical basis, is the antigenic and serological profile, which was used to initially group TG and SFG^22,23^. The antigens of TG and SFG were identified early on, starting in the early 70s, particularly the large, immunodominant surface proteins pan-*Rickettsia* OmpB and SFG, and the TRG-specific OmpA, which together make up the main Rickettsial S-layer ^46–51^. Congruent with phylogenetic taxonomy, the cell surface antigenic composition of SFG and TRG is the most closely related, with TG diverging before the split of TRG and SFG, hence lesser antigen identity, and lastly AG having split off in the early stages of rickettsial evolution, thus the reason for their lack of shared antigenic profile and non-pathogenicity. The primary proteins involved in immunogenicity, virulence, and infection belong to the Sca family and are found in most *Rickettsia* spp. such as Sca1, Sca2, Sca3, and Sca4^52–54^. Other surface-exposed proteins with reports of antigenicity include GroEL^52,55–58^, GroES^58^, Adr1 and Adr2, OmpW, Porin-4^58–60^, while other proteins with disputed or unknown antigenicity include TolC, TRG and SFG-specific RickA^56,61,62^. Apart from intra-genus antigenic composition variation, intra-species variation is also observed in strains and subspecies with different virulence, where specific epitopes are unrecognisable by monoclonal antibodies, suggesting that, despite shared antigenic composition, different strains and subspecies of the same species may not be antigenically identical and may be structurally different^63^. In which case, empirical confirmation is crucial for determining the antigenic profile shared between species, given new taxonomic resolution and existing antigenic or serologic research. This process is necessary to reconcile our previous serologic understanding with modern genomic data and taxonomic classification.

## Serological diagnosis and genomic correlation

### Historical overview of serological diagnosis in rickettsiae

Historically, the now-obsolete Weil-Felix test was the first rickettsial diagnostic technique used to diagnose typhus fever in 1916, utilising antigenic cross-reactivity between *Proteus* and *Rickettsia* species^64^. As diagnostic methods for rickettsial infections improved, techniques such as complement fixation and microagglutination replaced the Weil-Felix test^9,65^. With the advent of modern serological diagnosis, the immunofluorescence assay (IFA) emerged as the gold standard for rickettsial diagnosis due to its high accuracy^9,10^. Standard practice is to compare convalescent and acute-phase samples to detect a fourfold rise in antibody titer and confirm positivity^66–69^. Other serological techniques are still used in clinical settings, including enzyme-linked immunosorbent assay (ELISA), indirect immunoperoxidase (IIP), indirect hemagglutination (IHA), microagglutination (MA), and microimmunofluorescence (MIF).

### Antigenic profiling and early rickettsial classification

Serotyping, antigenic profiling, and cross-reactivity determination were largely conducted using MIF, IHA, MA, and IFA, which were important components of historical rickettsial classification. Early on, TG and SFG were classified using such methods, with a clear divide in antigenic distance, which forms the basis of modern group-based serological diagnosis^22,70^. A large part of rickettsial serology is the widespread, strong in-group cross-reactivity and demonstrable cross-reactivity across groups, particularly between TRG and SFG, TG and SFG, and within SFG^71–77^. As such, species-level identification is only possible with specialised techniques available only in a reference laboratory^9,10^.

### Genomic-antigenic correlations within the spotted fever group (SFG)

Within the spotted fever group, historical antigenic profiling shows strong concordance with modern WGS-based phylogenetic clustering. A side-by-side comparison between past antigenic relatedness and our current understanding of WGS rickettsial taxonomy provides a comprehensive overview that combines both fields into a cohesive big picture. Congruent with established WGS-based taxonomy, antigenic profile and serologic cross-reactivity show clear TG, TRG, and SFG separation, with SFG demonstrating clusters of distinct subgroups. The core SFG *R. rickettsii* subgroup is characterised by extensive antigenic similarity and cross-reactivity, including *R. rickettsii*, *R. conorii*, *R. africae*, *R. slovaca*, *R. parkeri*, and *R. sibirica* (Table 3). *R. sibirica* and *R. parkeri* are antigenically very similar, with a specificity difference (SPD) of 3.5, where < 3 SPD is considered the same species^22^, a finding that was later validated by Diop et al. and Chung et al. Within the *R. rickettsii* subgroup, *R. sibirica*, *R. conorii*, *R. rickettsii*, and *R. parkeri* form a strong network of serologically cross-reactive clusters of rickettsiae^22,76,78^. Recognition of *R. rickettsii* subsp. *californica* (formerly *R. philipii*, previously known as *Rickettsia* sp. 364D) is further supported by high cross-reactive IgG titres to *R. rickettsii*^79^, which was subsequently reclassified as *R. rickettsii* subsp. *californica* according to Diop et al.’s criteria^80^. Other serotyping characterisation methods, such as monoclonal antibodies, support the *R. slovaca* cluster with *R. africae* and *R. honei*, and other core SFG *R. rickettsii* subgroup rickettsiae.

**Table 3:** Table summarising antigenic cross-reactivity in *Rickettsia* groups. Cross-reactivity strength is broadly classified based on the principle of self cross-reactivity yielding the maximum signal which serves as a reference. Signals within similar range are classified as “strong”; notable signal drop but still significant is classified as “moderate”, while low to none signal is classified as “weak/limited”.

Phylogenetic Clade	Rickettsia Groups	Strong cross-reactivity	Moderate cross-reactivity	Limited/weak cross-reactivity
SFG	Core SFG *R. rickettsii* subgroup	*R. sibirica*, *R. conorii* complex, *R. rickettsii*, *R. parkeri. R. africae*, *R. honei*	*R. massiliae* subgroup *(R. massiliae*, *R. rhipicephali*, *R. montanensis*, *R. amblyommatis*, *R. aeschlemanii)*, *R. japonica/R. heilongjiangensis* subgroup (*R. japonica*), *R. helvetica* subgroup (*R. helvetica*), TRG	TG, AG
	*R. japonica/R. heilongjiangensis* subgroup	Core SFG *R. rickettsii* subgroup (*R. honei*, *R. rickettsii*)	Broad SFG, TRG	TG, AG
	*R. helvetica* subgroup	-	Broad SFG, TRG	TG, AG
	*R. tamurae* subgroup	-	Broad SFG, TRG	TG, AG
	*R. massiliae* subgroup	-	Core SFG *R. rickettsii* subgroup, Broad SFG, TRG (*R. felis*, *R. australis*)	TG, AG
TRG		*TRG (R. akari*, *R. australis*, *R. felis*, *R. tillamookensis)*, *R. massiliae* subgroup *(R. massiliae*, *R. montanensis)*	Broad SFG, TG	AG
TG		*R. prowazekii*, *R. typhi*	-	SFG, TRG, AG
AG		*-*	-	Purportedly TG

### Cross-reactivity beyond core SFG subgroups

Expanding from the core SFG group, intra-SFG rickettsiae continue to exhibit significant cross-reactivity across different subgroups, albeit at a limited degree compared to members of the same subgroup. *R. rickettsii* shows cross-reactivity to rickettsiae of the *R. massiliae* subgroup *R. massiliae*, *R. rhipicephali*, and *R. montanensis* in agreement with their taxonomic placement peripheral to the core group^79,81^. Monoclonal antibodies against *R. slovaca* similarly show limited cross-reactivity with *R. massiliae*, *R. rhipicephali*, *R. montanensis*, and *R. aeschlimannii*, all rickettsiae of the *R. massiliae* subgroup, as well as with *R. japonica* (or *R. heilongjiangensis* per the proposed reassignment) of the *R. heilongjiangensis* subgroup and *R. helvetica* of the *R. helvetica* subgroup^82^. Other evidence of the limited but well-observed cross-reactivity between the core *R. rickettsii* subgroup and *R. massiliae* subgroup includes *R. parkeri* observed cross-reactivity to *R. amblyommatis* by IFA^83^. While *R. japonica* has also demonstrated cross-reactivity with *R. rickettsii and R. honei*, it shares an antigenic profile with the *R. heilongjiangensis* subgroup and the core *R. rickettsii* subgroup^84^.

### The transitional group: Bridging serology and phylogeny

Expanding into TRG, the same congruent trend between past serotyping data and current WGS taxonomy is similarly notable. Among *R. felis*, *R. akari*, and *R. australis*, a strong antigenic relationship is observed as well as apparent taxonomic relatedness without the same taxonomic confusion that currently plagues SFG^84^. The TRG has come a long way and is quite established in modern rickettsiology. However, it is fascinating that antigenic profiling reported phylogenetics on this same grouping long before genetics-based approaches were popularised. Cementing TRG’s place as a closely diverged neighbour to SFG is documented cross-reactivity among the two groups. *R. felis* is shown to react with *R. montanensis* and *R. massiliae*^84^. Mouse serotyping of *R. australis* also indicates a close antigenic relationship to *R. montanensis*^22^. One reason for this specific closeness could be explained by their relative positions in their WGS phylogeny, where the *R. massiliae* subgroup diverged relatively early from the core SFG, which is directly preceded by the separation of TRG from the main SFG lineage^5,8,35^. More broadly towards the entire SFG, all three TRG rickettsiae display differing degrees of cross-reactivity to several and presumably all SFG rickettsiae^22^. *R. akari*, *R. australis*, *R. felis*, and even *R. tillamookensis* had been observed to cross-react with the SFG core *R. rickettsii* subgroup rickettsiae, such as with *R. akari*, *R. australis* to *R. honei*, *R. rickettsii*, *R. parkeri*, *R. slovaca*, *R. conorii*, *R. sibirica* and *R. tillamookensis* with *R. rickettsii*^22,81,82,84^. Functional cross-protection in *R. conorii* and *R. australis* supports antigenic similarity^85^.

### The typhus group (TG): Serological distance and diagnostic specificity

Antigenic profiling is much more straightforward in TG and AG due to their antigenic distance and the small number of species. *R. prowazekii* and *R. typhi* have long been grouped in the early days based on the similarity of antigenic profile in serotyping^22,84^. Literature support for TG-relatedness is extensive across research on serotyping and antigenic profiling. TG antigenic distance from other rickettsial groups also became abundantly clear towards all three SFG, TRG, and AG, due to early separation before the TRG SFG split. Despite being closely related yet distinct, TG still shares antigenic identity through surface proteins, now known to be mainly OmpB and Sca family proteins, which retroactively explains the observable yet minimal cross-reactivity between TG and other rickettsiae. Reports of the extent of the SFG TG cross-reactivity vary across the literature, with some reporting almost no cross-reactivity, such as between *R. prowazekii* and *R. rickettsii* in patients or with MIF^81,86^. Others report a more extensive shared antigenic relationship^84^. *R. typhi* and *R. japonica* cross-reactivity indicates that a degree of cross-group reactivity does indeed exist^75^. Interestingly, there appears to be a lower degree of shared antigenic profile between TRG and TG^22,84^. This division of TG into SFG and TG is reflected in modern serological diagnostic practice, where the preferred crude antigen selection includes SFG and TG rickettsiae to enable broad, group-specific pan-rickettsial diagnosis.

### The ancestral group (AG): Antigenic and genomic outliers

Lastly, AG has undergone extensive evolution distinct from the core rickettsial family, so that cross-reactivity is insignificant, if at all. AG antigenic distance is exacerbated by its evolutionary placement as the basal lineage of all rickettsiae. AG apparently does not share a clear antigenic profile with SFG or TRG, although there exists evidence supporting weak cross-reactivity with TG^82,84^. Unfortunately, because they are non-pathogenic and thus of lesser interest, there is limited research on their cross-reactivity with other rickettsiae. Interestingly, *R. canadensis* was once grouped into TG, likely due to weak cross-reactivity with *R. typhi*, *R. prowazekii*, and *R. belli*^84,86^.

## Strengths, limitations, and gaps in research

Serological methods and antigenic characterisation offer several advantages that bolster their viability and support the key characterisation of a polyphasic approach. They have long-standing historical support across various methodologies that have been extensively compared and tested, supplementing the investigation of clinical manifestations and pathogenesis. This serves as another pillar in the ideal polyphasic approach, a framework specifically tailored to the challenges unique to obligate intracellular bacteria such as *Rickettsia*^5,87^, and provides new insights that could benefit the development of vaccine and serodiagnosis tools.

Genomic and antigenic traits are intertwined by an inherent genotypic-phenotypic link; to consider one without the other is to ignore a large part of what constitutes a bacterial species. This interrelatedness can be explained mechanistically, as antigens are coded from bacterial genes, which serve as its blueprint. Antigenic epitopes cannot be visualised through genetic information alone, nor does it explain an evolutionary big picture. Any mutation in the genotype can drastically affect the phenotype over thousands of years of evolution and selection pressure, as is the case with *Rickettsia*, *whose* reductive evolution gives rise to more virulent species^87^. OmpA and OmpB are under intense positive natural selection pressure^88^, but the clinical and antigenic significance of the resulting antigenic diversity can only be conclusively determined by serological testing. Consequently, serotyping and antigenic profiling could serve a unique function as a valuable threshold metric to determine significant phenotypic differences between *Rickettsia* species when quantitative genomic thresholds fail to yield adequate resolution to species delineation in the face of closely related species such as *R. parkeri* and *R. slovaca*, as well as to account for future species designation and revised species delineation. Importantly, concordance between antigenic and genomic relationships is not universal, and antigenic data should be interpreted as complementary rather than definitive evidence of phylogenetic relatedness.

Antigenic data were widely used for a long time prior to the development of molecular and genomic frameworks. Mouse serotyping was the gold standard for antigenic profiling and played a crucial role in the early classification of TG and SFG^22,86^. Even in the present, serological assays still see wide usage in the diagnosis of *Rickettsia* infections. However, serological assays are being phased out as molecular and genomic methods come to prominence. Due to the limited scope of this review, a practical antigenic-genomic framework poses challenges that are difficult to address. Serological methodologies have been used historically, all of which share the same pitfalls in serological assays: a lack of cut-off standardisation, methodological variability, varying degrees of subjective interpretation, and, most importantly, the fact that many serological methods have not yet been standardised in the context of *Rickettsia* diagnosis and cross-reactivity. Discussions should also be had about which classical serological assays is still rigorous and which is obsolete^89^, with tests such as the Weil Felix test now widely considered obsolete for its poor sensitivity and specificity despite its ability to differentiate SFG and TG^64,90^. Although there is no formal standardisation guideline, general agreement exists among serological classifications, which are supported by an immunological basis^22^. Additionally, serological antigen preparation is complicated by the fact *Rickettsia* are biosafety level 3 pathogens, hampering the accessibility of these pathogens to laboratories with resources and appropriate facilities^7^. Serological assays inherently yield lower phylogenetic resolution than modern genomic tools due to reliance on semi-quantitative titers and subjective interpretation, whereas DNA is not subject to immunological variation and is more reproducible.

Beyond serological classification, the unresolved classification issue has also been exacerbated by the use of multigenic sequencing approaches^7,34^. As noted by Dumler and Walker, the widely adopted Fournier multigenic scheme was developed with cutoffs based on mouse serotyping. Serological classification is not adequate to ascertain phylogenetic relationships on its own due to lower clarity and phylogenetic resolution than molecular methods. As such, calibrating a multigenic approach back to mouse serotyping results in a threshold too lax, which, according to Dumler and Walker, is repeated by Diop et al. when they propose their genomic framework against the prior multigenic scheme^7,8^. To that end, for a true polyphasic approach, genomic taxonomy should form the foundation of ongoing future *Rickettsia* taxonomic revisions, with serological data supplementing essential characterisation of species, from antigenic relationships to clinical relevance and phenotypic characteristics. Instead, if old serological frameworks are problematic, then new frameworks should be revised to bridge phenotype to genotype.

Although this review proposes a polyphasic framework integrating genomic and antigenic evidence, the framework remains conceptual. We do not propose validated quantitative cut-offs, decision rules, or practical guidelines for integrating whole-genome sequencing and serological or antigenic evidence into taxonomic decision-making. Developing and validating such criteria will require systematic comparative datasets, broader consensus within the field, and prospective evaluation. Accordingly, establishing an operational polyphasic framework represents an important direction for future research.

Dumler and Walker also argue that another important phenotypic attribute to consider is pathogenicity, which has made little progress in research^7^. To that end, we agree with the importance of polyphasic approaches to current species delineation guidelines. Antigenic profiling is an important phenotypic attribute that should not be overlooked, despite its historical origins and fading popularity in the genomic era. In the case of various *Rickettsia* species with virulent and avirulent strains, the antigenic profile may play a large role in differences in pathogenicity, as in *R. rickettsii* Sheila Smith and *R. rickettsii* Iowa^91^. Ultimately, it is imperative that bacterial characteristics such as antigenic profile, geographical, chemotaxonomic, and phenotypic traits supplement genomic understanding of *Rickettsia* taxonomy for a comprehensive characterisation.

## Conclusion

This review suggests that historical antigenic profiling of *Rickettsia* captures biologically meaningful relationships that are broadly concordant with many relationships recovered by whole-genome sequencing-based phylogeny. Far from being obsolete, these serological data anticipated many of the groupings now recovered by genome-wide analyses and provide an independent line of evidence supporting the proposed phylogenetic structure in this review.

As had been demonstrated, classical molecular and gene-based metrics alone are insufficient to resolve *Rickettsia* taxonomy in a biologically coherent manner. The obligate intracellular lifestyle, limited phenotypic diversity and low genomic divergence of *Rickettsia*, both between and within species, challenge the application of standard bacterial species thresholds, resulting in taxonomic instability and inconsistent species delineation when classical genetic classification is used in isolation. While genomic-based metrics can reliably elucidate *Rickettsia* taxonomic relationships and should remain the preferred foundation of phylogenetic resolution, existing conventional species demarcation criteria raise questions about the appropriate species threshold and whether an ideal threshold can even be determined. Given the explosion in genome sequencing resulting in the discovery of many previously unknown species, including many rickettsial endosymbionts, which are known only from molecular evidence, a metric based on genetic material alone is valuable to determine the taxonomic classification of these novel species, and molecular classification is increasingly used in large-scale bacterial genome analysis. However, this must be balanced with the use of more clinically relevant classifications for pathogenic species, as these molecular schemes are designed to improve the classification of uncultured species and may not be informative for pathogenic organisms^92^. Given the large number of SFG species which are highly genetically related and do not meet the genetic definitions of a species, a classification scheme which focuses on sub-groups within the wider SFG clade may be more useful than an effort to delineate exact species boundaries and may inform a revised approach to the definition of novel SFG species.

Future work should focus on developing and validating practical polyphasic frameworks that integrate genomic and antigenic evidence for species delineation, diagnostic antigen selection and future taxonomic proposals. Such an approach has direct implications for rational species designation, selection of reference antigens for serological assays, and the development of improved diagnostic tools and vaccines. Re-establishing antigenic data as a complementary component of rickettsial taxonomy is therefore not a regression but a necessary step toward a coherent, biologically meaningful, and clinically relevant framework that complements genome-based classification in a genus where classical species concepts fail.

## Supplementary information

Supplementary Tables S1, S2.

## Data Availability

No datasets were generated or analysed during the current study.
