# Supplementary material for: Bridging historical antigenic profiling and whole-genome taxonomy in Rickettsia
Source: Sci Rev. 2026 Sep 14;1(1):8. doi: 10.1038/s44573-026-00004-2 (PMC13602854; doi:10.1038/s44573-026-00004-2)
Supplement: Supplementary file 1 [file 44573_2026_4_MOESM1_ESM.docx]

**Table S1**: A summary of publicly available *Rickettsia* genomes. Data on all *Rickettsia* genomes were downloaded from NCBI Datasets, excluding those assembled solely from metagenomes, and grouped by species as assigned by NCBI. Substrains were included with the parent species, while all subspecies were counted separately.

| **Species** | **Assembly level** | | | |  |
| --- | --- | --- | --- | --- | --- |
|  | **Complete Genome** | **Chromosome** | **Scaffold** | **Contig** | **Total** |
| Candidatus Rickettsia barbarica |  |  |  | 1 | 1 |
| Candidatus Rickettsia colombianensi |  |  |  | 1 | 1 |
| Candidatus Rickettsia kedanie |  |  |  | 1 | 1 |
| Candidatus Rickettsia kotlanii | 2 |  |  |  | 2 |
| Candidatus Rickettsia mendelii |  |  | 3 | 2 | 5 |
| Candidatus Rickettsia senegalensis |  |  |  | 1 | 1 |
| Candidatus Rickettsia tasmaniensis |  |  |  | 1 | 1 |
| Candidatus Rickettsia Torix (Bemisia tabaci) |  |  | 1 |  | 1 |
| *Rickettsia aeschlimannii* |  |  |  | 3 | 3 |
| *Rickettsia amblyommatis* | 3 |  | 1 | 2 | 6 |
| *Rickettsia argasii T170-B* |  |  |  | 1 | 1 |
| *Rickettsia asembonensis* |  |  |  | 1 | 1 |
| *Rickettsia asiatica* | 1 |  |  |  | 1 |
| *Rickettsia bellii* | 3 |  |  | 2 | 5 |
| *Rickettsia conorii subsp. caspia A-167* |  |  |  | 1 | 1 |
| *Rickettsia conorii subsp. heilongjiangensis* | 6 |  | 1 |  | 7 |
| *Rickettsia conorii subsp. indica ITTR* |  |  |  | 1 | 1 |
| *Rickettsia conorii subsp. israelensis ISTT CDC1* |  |  |  | 1 | 1 |
| *Rickettsia conorii subsp. raoultii* | 3 | 2 |  | 1 | 6 |
| *Rickettsia endosymbiont of Aspidapion aeneum* | 1 |  |  |  | 1 |
| *Rickettsia endosymbiont of Cantharis rufa* | 1 |  |  |  | 1 |
| *Rickettsia endosymbiont of Cardiosporidium cionae* |  |  |  | 1 | 1 |
| *Rickettsia endosymbiont of Ceutorhynchus assimilis* |  | 1 |  |  | 1 |
| *Rickettsia endosymbiont of Ceutorhynchus obstrictus* | 1 |  |  |  | 1 |
| *Rickettsia endosymbiont of Culicoides newsteadi* |  |  | 1 |  | 1 |
| *Rickettsia endosymbiont of Gonocerus acuteangulatus* | 1 |  |  |  | 1 |
| *Rickettsia endosymbiont of Halotydeus destructor* |  |  |  | 1 | 1 |
| *Rickettsia endosymbiont of Hedychridium roseum* | 1 |  |  |  | 1 |
| *Rickettsia endosymbiont of Ixodes pacificus* |  |  |  | 1 | 1 |
| *Rickettsia endosymbiont of Ixodes scapularis* |  | 1 |  |  | 1 |
| *Rickettsia endosymbiont of Lasioglossum villosulum* | 1 |  |  |  | 1 |
| *Rickettsia endosymbiont of Nabis limbatus* | 1 |  |  |  | 1 |
| *Rickettsia endosymbiont of Oedothorax gibbosus* | 1 |  |  |  | 1 |
| *Rickettsia endosymbiont of Orchestes rusci* |  |  |  | 1 | 1 |
| *Rickettsia endosymbiont of Pantilius tunicatus* | 1 |  |  |  | 1 |
| *Rickettsia endosymbiont of Polydrusus tereticollis* | 1 |  |  |  | 1 |
| *Rickettsia endosymbiont of Proechinophthirus fluctus* |  |  | 1 |  | 1 |
| *Rickettsia endosymbiont of Rhinocyllus conicus* | 1 |  |  |  | 1 |
| *Rickettsia endosymbiont of Seladonia tumulorum* | 1 |  |  |  | 1 |
| *Rickettsia endosymbiont of Thecocarcelia acutangulata* | 1 |  |  |  | 1 |
| *Rickettsia endosymbiont of Urophora cardui* |  |  |  | 1 | 1 |
| *Rickettsia endosymbiont of Villa modesta* | 1 |  |  |  | 1 |
| *Rickettsia felis* | 1 |  |  | 4 | 5 |
| *Rickettsia fournieri* |  |  | 1 |  | 1 |
| *Rickettsia helvetica* | 2 | 1 |  |  | 3 |
| *Rickettsia hoogstraalii* |  |  | 1 | 2 | 3 |
| *Rickettsia japonica* | 15 | 20 |  | 1 | 36 |
| *Rickettsia koreansis* |  |  |  | 1 | 1 |
| *Rickettsia lanei* | 1 |  |  |  | 1 |
| *Rickettsia monacensis* | 1 |  |  |  | 1 |
| *Rickettsia oklahomensis* | 1 |  |  |  | 1 |
| *Rickettsia parkeri* | 4 |  |  | 3 | 7 |
| *Rickettsia prowazekii* | 9 | 1 |  | 2 | 12 |
| *Rickettsia rhipicephali* | 2 |  | 1 | 1 | 4 |
| *Rickettsia rickettsii* | 26 | 1 |  |  | 27 |
| *Rickettsia sibirica* | 10 |  |  | 1 | 11 |
| *Rickettsia sibirica subsp. mongolitimonae HA-91* |  |  |  | 1 | 1 |
| *Rickettsia sp. 109562A South Dakota* | 1 |  |  |  | 1 |
| *Rickettsia sp. 2024-CO-Wats* | 1 |  |  |  | 1 |
| *Rickettsia sp. 2024-CO-Wats-2* | 1 |  |  |  | 1 |
| *Rickettsia sp. MAE-05* |  |  |  | 1 | 1 |
| *Rickettsia sp. MEAM1 (Bemisia tabaci)* | 1 |  |  | 1 | 2 |
| *Rickettsia sp. R1* |  |  | 1 |  | 1 |
| *Rickettsia sp. Tenjiku01* |  |  |  | 1 | 1 |
| *Rickettsia sp. TH2014* |  |  | 1 |  | 1 |
| *Rickettsia sp. wb* |  |  |  | 1 | 1 |
| *Rickettsia sp. wq* |  |  |  | 1 | 1 |
| *Rickettsia tamurae* |  |  |  | 1 | 1 |
| *Rickettsia tamurae subsp. buchneri* |  |  |  | 1 | 1 |
| *Rickettsia tillamookensis* | 1 |  |  |  | 1 |
| *Rickettsia typhi* | 3 |  |  |  | 3 |
| *Rickettsia conorii* | 1 |  |  |  | 1 |
| *Rickettsia australis* | 1 |  |  | 1 | 2 |
| *Rickettsia canadensis* | 2 |  |  |  | 2 |
| *Rickettsia akari* | 1 |  |  |  | 1 |
| *Rickettsia honei* |  |  |  | 1 | 1 |
| *Rickettsia gravesii* |  |  |  | 1 | 1 |
| *Rickettsia prowazekii* | 1 |  |  |  | 1 |
| *Rickettsia sibirica subsp. sibirica* | 1 |  |  |  | 1 |
| *Rickettsia massiliae* | 1 | 1 |  |  | 2 |
| *Rickettsia montanensis* | 1 |  |  |  | 1 |
| *Rickettsia africae* | 1 |  |  |  | 1 |
| *Rickettsia slovaca* | 2 |  |  |  | 2 |
| *Rickettsia typhi* | 1 |  |  |  | 1 |
| *Rickettsia peacockii* | 1 |  |  |  | 1 |
| *Rickettsia rickettsii* subsp. *californica* | 1 |  |  |  | 1 |

**Table S2**. Reference genomes of *Rickettsia* strains used in this study, including accession numbers and relevant metadata for each genome included in the average nucleotide identity (ANI) analysis in Figure 4.

| **Species** | **Strain ID** | **Assembly accession** |
| --- | --- | --- |
| *Rickettsia aeschlimannii* | MC16 | GCF_001051325.1 |
| *Rickettsia africae* | ESF-5 | GCF_000023005.1 |
| *Rickettsia akari* | Hartford | GCF_000018205.1 |
| *Rickettsia amblyommatis* | Ac37 | GCF_001273795.1 |
| *Rickettsia argasii* | T170-B | GCF_000965185.1 |
| *Rickettsia asembonensis* | NMRCii | GCF_000828125.2 |
| *Rickettsia asiatica* | Maytaro1284 | GCF_007989425.1 |
| *Rickettsia australis* | Cutlack | GCF_000284155.1 |
| *Rickettsia bellii* | RML369-C | GCF_000012385.1 |
| *Rickettsia buchneri* | PalLab | GCF_026723685.1 |
| *Rickettsia canadensis* | CA410 | GCF_000283915.1 |
| *Rickettsia conorii* | Malish 7 | GCF_000007025.1 |
| *Rickettsia felis* | LSU-Lb | GCF_000804505.1 |
| *Rickettsia fournieri* | AUS118 | GCF_900243065.1 |
| *Rickettsia gravesii* | BWI-1 | GCF_000485845.1 |
| *Rickettsia heilongjiangensis* | CH8-1 | GCF_009731545.1 |
| *Rickettsia helvetica* | OB144 | GCF_963970025.1 |
| *Rickettsia honei* | RB | GCF_000263055.1 |
| *Rickettsia japonica* | YH | GCF_000283595.1 |
| *Rickettsia massiliae* | MTU5 | GCF_000016625.1 |
| *Rickettsia monacensis* | IrR/Munich | GCF_000499665.2 |
| *Rickettsia montanensis* | OSU 85-930 | GCF_000284175.1 |
| *Rickettsia parkeri* | Atlantic Rainforest | GCF_005549115.1 |
| *Rickettsia peacockii* | Rustic | GCF_000021525.1 |
| *Rickettsia prowazekii* | Chernikova | GCF_000277165.1 |
| *Rickettsia raoultii* | BIME | GCF_023674445.1 |
| *Rickettsia rhipicephali* | 3-7-female6-CWPP | GCF_000284075.1 |
| *Rickettsia rickettsii* | Sheila Smith | GCF_000018225.1 |
| *Rickettsia sibirica* | 246 | GCF_000166935.1 |
| *Rickettsia slovaca* | 13-B | GCF_000237845.1 |
| *Rickettsia tamurae* | AT-1 | GCF_000751075.1 |
| *Rickettsia tillamookensis* | Tillamook 23 | GCF_016743795.2 |
| *Rickettsia typhi* | Wilmington | GCF_000008045.1 |
